# A dual-channel optogenetic stimulator selectively modulates distinct defensive behaviors

**DOI:** 10.1016/j.isci.2021.103681

**Published:** 2021-12-24

**Authors:** Xue Cai, Lizhu Li, Wenhao Liu, Nianzhen Du, Yu Zhao, Yaning Han, Changbo Liu, Yan Yin, Xin Fu, Dawid Sheng, Lan Yin, Liping Wang, Pengfei Wei, Xing Sheng

**Affiliations:** 1 Department of Electronic Engineering, Beijing National Research Center for Information Science and Technology, Center for Flexible Electronics Technology, and IDG/McGovern Institute for Brain Research, Tsinghua University, Beijing 100084, China; 2 Shenzhen Key Lab of Neuropsychiatric Modulation and Collaborative Innovation Center for Brain Science, Guangdong Provincial Key Laboratory of Brain Connectome and Behavior, CAS Center for Excellence in Brain Science and Intelligence Technology, Brain Cognition and Brain Disease Institute (BCBDI), Shenzhen Institutes of Advanced Technology, Chinese Academy of Sciences, Shenzhen-Hong Kong Institute of Brain Science, Shenzhen Fundamental Research Institutions, Shenzhen 518055, China; 3 Department of Biomedical Sciences, City University of Hong Kong, Kowloon Tong, Hong Kong 999077, China; 4 University of the Chinese Academy of Sciences, Beijing 100049, China; 5 School of Materials Science and Engineering, Hangzhou Innovation Institute, Beihang University, Beijing 100191, China; 6 School of Materials Science and Engineering, Tsinghua University, Beijing 100084, China

**Keywords:** Optoelectronics, Neuroscience, Bioelectronics

## Abstract

Implantable devices and systems have been emerging as powerful tools for neuroscience research and medical applications. Here we report a wireless, dual-channel optoelectronic system for functional optogenetic interrogation of superior colliculus (SC), a layered structure pertinent to defensive behaviors, in rodents. Specifically, a flexible and injectable probe comprises two thin-film microscale light-emitting diodes (micro-LEDs) at different depths, providing spatially resolved optical illuminations within the tissue. Under remote control, these micro-LEDs interrogate the intermediate layer and the deep layer of the SC (ILSC and DLSC) of the same mice, and deterministically evoke distinct freezing and flight behaviors, respectively. Furthermore, the system allows synchronized optical stimulations in both regions, and we discover that the flight response dominates animals' behaviors in our experiments. In addition, c-Fos immunostaining results further elucidate the functional hierarchy of the SC. These demonstrations provide a viable route to unraveling complex brain structures and functions.

## Highlights

• A wireless implant with two micro-LEDs enables dual-channel optogenetic stimulations
• Two micro-LEDs stimulate the intermediate and the deep layers of superior colliculus
• Dual-channel stimulations selectively evoke suppressed or promoted moving behaviors
• Synchronized stimulations in the intermediate and the deep layers are achieved

## Graphical abstract

**Figure:**
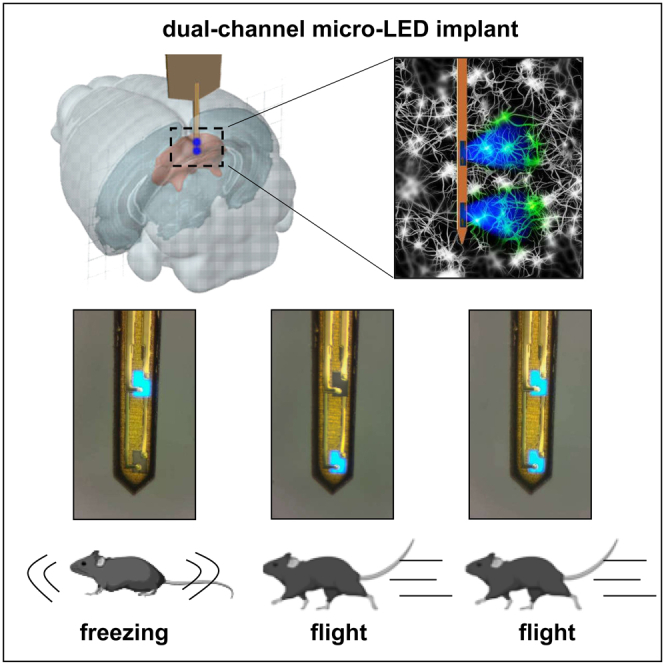

## Introduction

Optogenetics has been emerging as an essential tool in neuroscience research in recent years, owing to its cell specific modulation capability with desirable temporal and spatial resolutions (Deisseroth, 2011; Yizhar et al., 2011). Optical fibers based on glass and other dielectric materials (Park et al., 2017; Ung and Arenkiel, 2012; Wang and Dong, 2020), which are tethered with external sources, deliver light signals into targeted brain regions expressing corresponding opsins for designed neural activation or inhibition. Most conventional fibers, however, lack the ability to distribute photons among multiple brain regions. Exploited techniques based on fiber bundles, patterned tapered fibers, and lithographically defined waveguides (Farah et al., 2015; Nazempour et al., 2020; Pisanello et al., 2015, 2018) can spatially address different brain regions, but most of them are still constrained by wiring, which limits the exploration of animals' freely moving behaviors. Recently developed microsized, cellular scale optoelectronic devices, in synergy with the miniaturized wirelessly operated circuits, provide a viable solution for interfacing with the nervous system, supplying versatile functions such as optogenetic interrogation, optoelectrochemical recording, and pharmacology (Gutruf et al., 2018; Jeong et al., 2015; Kim et al., 2013; Liu et al., 2020; Montgomery et al., 2015; Shin et al., 2017; Zhang et al., 2019; Zhao et al., 2019; Mayer et al., 2019). Existing works include the utilization of multi-channel, microscale light-emitting diodes (micro-LEDs) for applications in the rodent brain, cochlea, and spinal cord (Ayub et al., 2017; Keppeler et al., 2020; Park et al., 2015; Rossi et al., 2015; Yang et al., 2021). With these efforts being attempted, we envision that these microscale devices, with independently addressable stimulating and recording features, can further help unravel the structure and function of the nervous system when implanted in the deep brain.

One example of complex brain regions is the superior colliculus (SC), which processes and mediates innate defensive behaviors and plays critical roles in animals' survival in natural environment (De Franceschi et al., 2016; Dean et al., 1989; May, 2006; Oliveira and Yonehara, 2018). The rodent medial SC comprises layered structures including the superficial, the intermediate, and the deep layers (SLSC, ILSC, and DLSC) at various depths (May, 2006; Wei et al., 2015; Zhou et al., 2019). Specifically, SLSC includes stratum zonale, stratum griseum superficiale, and stratum opticum, ILSC includes stratum griseum intermedium and stratum album intermedium, and DLSC includes stratum griseum profundum and stratum album profundum (Gandhi and Katnani, 2011). Previous works discover that these different layers project through different pathways under different stimuli, thereby initiating distinct types of defensive behaviors including freezing and flight (Evans et al., 2018; Wei et al., 2015). These studies rely on fiber based optogenetic and photometric tools to regulate and record neural signals in different layers. However, existing techniques only interrogate with one single layer at a time, making it difficult to provide spatially resolved stimulations among these separate layers and trigger different behaviors. In addition, the vertically stacked layered structure of SC imposes significant challenges for stimulating one specific layer with fiber optics. Moreover, the tethered instrumentation may impose restrictions on animals' free movements and cause unwanted responses (Gutruf et al., 2018a; Kale et al., 2015). Finally, defensive behaviors of animals could be mutually exclusive, and animals can only show one type of behavior at one time, in response to various stimuli in a complex natural environment. Therefore, devices that are able to provide independent multi-sites optogenetic stimulations are urgently desired for behavioral choice studies.

In this paper, we investigate the defensive responses of freely moving mice by implementing a wireless, dual-channel optoelectronic probe interfaced with the SC. With two micro-LEDs assembled on a flexible substrate and operated via a wireless circuit, a needle-shaped implant can independently or simultaneously regulate two distinct layers. Demonstrated in the same animals, optogenetic stimulations generated in the ILSC and the DLSC elicit corresponding freezing and flight responses, respectively. Furthermore, the dual-channel probe allows simultaneous stimulation of the two layers, in which we identify that the flight response caused by stimulation of the DLSC dominates the animals' behaviors. In summary, such an optoelectronic neural interface provides a powerful means to understand the sophisticated structure-function relationship of the brain system.

## Results

### Wirelessly operated, dual-channel micro-LED probes, and circuit systems

**Figure 1:**
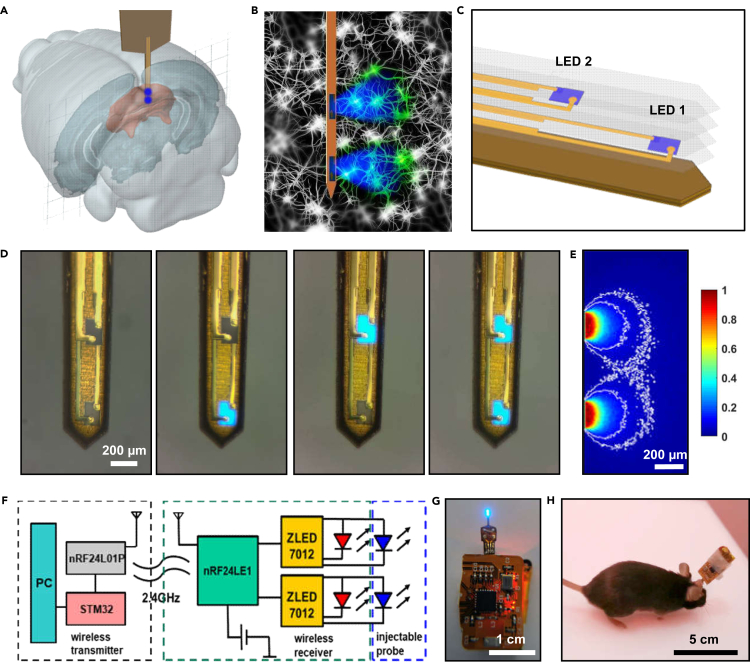
Wirelessly operated, dual-channel micro-LED probes for optogenetic control of neural activities in the superior colliculus (SC) (A) Schematic model showing the probe implanted in the medial SC of a mouse brain. (B) Conceptual illustration of the micro-LED probe for dual-channel optogenetic stimulation within the neural system. (C) Explosive view of the probe, comprising two blue micro-LEDs at different sites, with metalized electrodes, insulating, and encapsulating layers, based on a copper coated polyimide (Cu/PI/Cu) substrate. (D) Optical micrographs of a fully fabricated probe showing independent operations of the two micro-LEDs (injection current ∼0.1 mA). (E) Simulations of optical propagations from the micro-LEDs to the brain tissue. Iso-intensity lines show 10% and 5% of the maximum power. (F) Block diagram of the wireless circuit design. (G) Photograph of a probe powered by the wireless circuit. (H) A wireless module mounted on the head of a behaving mouse.

Figures 1A and 1B schematically illustrate the dual-channel probe implanted into the mouse medial SC, with two micro-LEDs, which independently address two separate layers (ILSC and DLSC). The explosive structure of the micro-LED probe is displayed in Figure 1C, with fabrication details provided in Figure S1 and the supplemental information. Two thin-film indium gallium nitride (InGaN) blue micro-LEDs (size: 180 μm × 125 μm × 7 μm, separation spacing 610 μm) are formed by laser liftoff and mount on a thin-film copper (Cu) and polyimide (PI) (10 μm PI/18 μm Cu/25 μm PI/18 μm Cu) based, needle-shaped substrate, followed by metallization and encapsulation (Li et al., 2018; Zhao et al., 2019). The Cu/PI/Cu substrate has a measured Young's modulus of ∼15 GPa, softer than silicon (∼180 GPa) and tungsten (∼400 GPa), but still much harder than the brain tissue (∼1 kPa). Encapsulated by a PDMS/parylene-C bilayer, the micro-LED probe can work properly for more than one month *in vivo* and *in vitro* (in an accelerated test in phosphate-buffered saline solution at 70°C), with negligible functional degradations (Zhao et al., 2019) and minimal leakage of Cu element into the brain tissue. Figure 1D shows the fabricated probe with a width of 310 μm and a thickness of 150 μm. The probe has a bending stiffness of ∼9 × 10^4^ pNm^2^, similar to metallic probes applied for *in vivo* electrophysiological recordings (He et al., 2020). The two micro-LEDs exhibit electroluminescence peaked at ∼470 nm, which can operate independently and stimulate channelrhodopsin-2 (ChR2) expressing neurons in separate layers of SC. Compared to conventional fiber optics, such a dual-channel micro-LED probe is advantageous to interrogate SC, because the lateral emission of the probe allows it to spatially resolve two separate layers, whereas the downward emission of a fiber can easily evoke both layers.

Figure 1E simulates optical power distributions for LED emissions within the brain tissue, based on a Monte-Carlo ray tracing method (Wang et al., 1995). Modeling results indicate that optical intensity attenuates to ∼10% and ∼5% of the irradiance on the device surface, at distances of 300 μm and 470 μm (Figures 1E and S2A), respectively. The simulated light distribution contour (Figure S2B) is similar to the measured results in a brain phantom (Figure S2C), though with differences because of the isotropy of brain phantom, and consistent with the literature (Yizhar et al., 2011). The measured power density of these blue micro-LEDs ranges from 10 mW/mm^2^ to 50 mW/mm^2^ at injection currents of 1–5 mA, which are similar to results reported in the literature and sufficient for optically exciting ChR2-expressing cells (Mayer et al., 2019; Yang et al., 2021; Keppeler et al., 2020; Kim et al., 2013) (Table S1). In addition, thermal measurements and models (Figure S3) performed in air and in the brain phantom predict that the maximum temperature rise associated with the micro-LED operation can be controlled within 1°C at currents of 1–5 mA in the brain. In addition, the maximum operating temperature of the circuit board is about 34°C tested on paper sheets, mostly concentrated on the Bluetooth chip and does not affect the temperature of the implant as well as the brain tissue (Figure S3C). Although it is ∼6°C higher than the room temperature, it is still lower than the mouse body temperature. In subsequent *in vivo* optogenetic experiments, the micro-LEDs operate at 5 mA so that the provided irradiance is sufficient for ChR2 activation (threshold power ∼1 mW/mm^2^) (Boyden et al., 2005; Degenaar et al., 2009), while minimizing the tissue heating and optical crosstalk between the ILSC and the DLSC at the same time.

A battery powered, wireless circuit (size, ∼19 × 12 mm^2^; weight, ∼1.7 g) controls the dual-channel micro-LED probe implanted into the mouse brain (Figure S4). The circuit design scheme and photograph are illustrated in Figures 1F–1H. Operated by a remote transmitter connected to a computer, the receiver circuit couples with the injected probe and mounts on the head of behaving mice, and the circuit controller is unplugged and disconnected from the probe when mice are not used for behavior tests. The emissive power, pulse frequencies, and widths of each blue micro-LED can be separately programmed in real time. In addition, the circuit hardware allows independent control of multiple receivers (Video S1), which are beneficial for studying complicated social behaviors among multiple animals.

Video S1. Video showing four micro-LED probes connected with wireless receiving circuits, operated in different modes independently. Related to Figure 1

### Alternated optogenetic stimulations in the ILSC and the DLSC elicit distinct defensive behaviors

**Figure 2:**
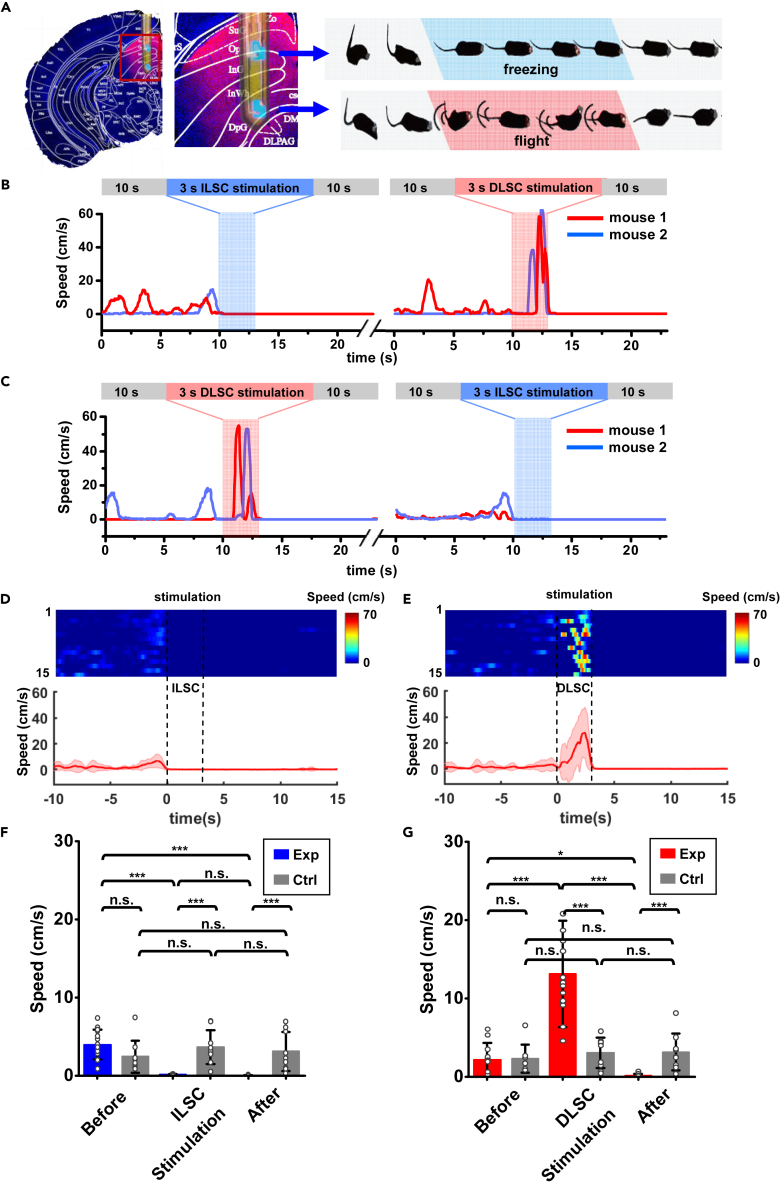
Analyses of defensive behaviors under alternating stimulations of intermediate and deep layers (ILSC and DLSC) with a wireless, dual-channel micro-LED probe implanted into the SC of mice (A) Coronal SC section overlaid with a micro-LED probe (left), and schemes of corresponding behaviors (freezing and flight) under stimulations (right). (B and C) Representative time courses of locomotion speed from two mice, with (B) a 3-s stimulation applied in the ILSC and another 3-s stimulation in the DLSC (∼5 min interval), and (C) vice versa. (D and E) Time courses of locomotion speed when stimulating (D) the ILSC and (E) the DLSC. Top: Heatmaps of 15 individual trials from 5 mice. Bottom: Averaged locomotion speeds versus time. The solid lines and shaded areas indicate the mean and s.e.m., respectively. (F and G) Quantitative analyses of locomotion speed before, during, and after optogenetic stimulations in (F) the ILSC and (G) the DLSC. Results are averaged (E) before (10 s), during ILSC (3 s), and after stimulations (10 s). (F) before (10 s), during DLSC (3 s), and after stimulations (10 s). The statistical analysis method is two-way repeated measures ANOVA (*n* = 15 trails for Exp and *n* = 9 trails for Ctrl, Sidak's multiple comparisons test, ∗∗∗p <0.001, ∗∗p <0.01, ∗p <0.05, n.s. p >0.05). Values are represented as mean ± s.d.

We inject adeno-associated virus (AAV) carrying ChR2 under the control CaMKII promoter (AAV-mCaMKIIa-hChR2-mCherry) to the SC of mice, and implant the dual-channel micro-LED probes 3 weeks post the virus injection (Figure 2A, left). With designed device spacing and emission intensity (Figure 1E), optical irradiances from the two micro-LEDs completely cover the ILSC (and partially the optical layer in SLSC) and the DLSC, which can potentially evoke freezing and flight behaviors in a same subject, respectively (Figure 2A, right). In previous studies (Stubblefield et al., 2015; Zurita et al., 2018), the optogenetic experiments were only performed in separated animals, because conventional silica fibers were limited by single site emissions. These thin-film flexible micro-LED probes cause similar tissue lesions as well as immune responses in comparison with conventional silica fibers with a diameter of 200 μm (Figure S5). 1 week after the probe implantation, we perform behavior tests on these animals by wirelessly controlling the two micro-LEDs. The micro-LEDs operate at a frequency of 20 Hz, a pulse width of 10 ms, an injection current of 5 mA and duration of 3 s. Optogenetic stimulations in the ILSC and the DLSC cause clearly reduced and enhanced movement, respectively (Videos S2 and S3). These behavioral responses are in agreement with previously reported “freezing” and “flight” behaviors (Evans et al., 2018; Wei et al., 2015). Moreover, the two micro-LEDs can be operated in a temporally separated mode, which evokes alternating freezing and flight behaviors. Figures 2B and 2C present example traces of recorded locomotion speeds from two mice in response to distinct stimulations by the two micro-LEDs, with an interval of ∼5 min. In these experiments, the choice of different defensive behaviors correlates with the stimulated brain regions and is not affected by the temporal stimulation sequences. It is also noted that the defensive behaviors (freezing or flight) are dependent on the testing history and attenuate after multiple repeated stimulations, which are associated with the complex neural pathways connected to the SC (Almada et al., 2018; Baek et al., 2019). Here we select the first 3 trails of each subject for statistical analysis. Figures 2D and 2E show more traces and averaged speed from multiple animals. Figures 2F and 2G compare statistical results between the experimental group (Exp) with ChR2 and the control group (Ctrl) without ChR2 (injecting AAV-mCaMKIIa-mCherry). Traces for the control group are shown in Figure S6A. Here we record the average locomotion speed 10 s before, 3 s during, and 10 s after optogenetic stimulations in the ILSC (Figure 2F) and the DLSC (Figure 2G), respectively. Although the normal behavior of mice provide an average speed of ∼5 cm/s, quantitative analysis reveals that the animal speed experiences a significant drop to nearly zero under stimulation in the ILSC. Furthermore, the freezing behavior persists for more than 10 s after the optical stimulation. On the other hand, the same experimental group displays significantly enhanced locomotion (with an average speed more than 10 cm/s) in response to stimulations in the DLSC. Similar to the behaviors after stimulations in the ILSC, animals also exhibit prolonged freezing responses after stimulations in the DLSC. These behaviors are in accordance with reports demonstrated with stimulations generated by fiber-optics based light sources and external environmental stimuli (Shang et al., 2015, 2018; Wei et al., 2015). By contrast, the control group shows no significant change in the locomotion, during and after stimulations in the ILSC or the DLSC.

Video S2. Representative video showing a freely behaving mouse implanted with the micro-LED probe and the head-mounted circuit, when providing optogenetic stimulation in the ILSC. LED current 5 mA, frequency 20 Hz, duty cycle 20%, and stimulation duration 3 s. Related to Figure 2

Video S3. Representative video showing a freely behaving mouse implanted with the micro-LED probes and the head-mounted circuit, when providing optogenetic stimulation in the DLSC. LED current 5 mA, frequency 20 Hz, duty cycle 20%, and stimulation duration 3 s. Related to Figure 2

### Synchronized optogenetic stimulations in the ILSC and the DLSC

**Figure 3:**
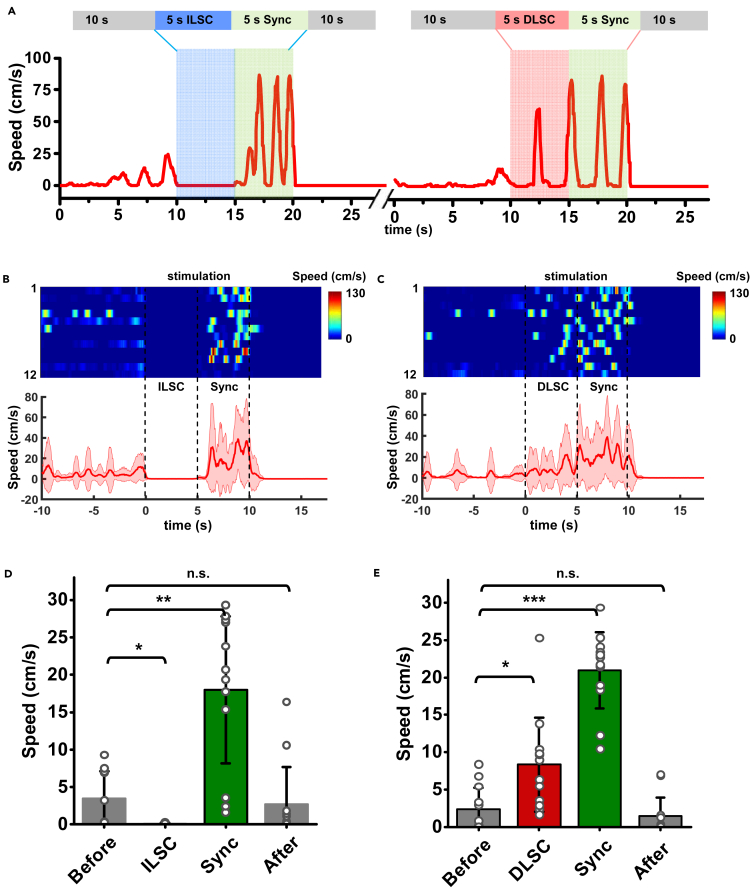
Analyses of defensive behaviors under stimulations of intermediate or deep layers (ILSC and DLSC) followed by synchronized stimulations in both sites (Sync) (A) Representative time courses of locomotion speed from a mouse, with a 5-s stimulation applied in the ILSC and the DLSC followed by another 5-s stimulation in both sites (Sync) alternately. Experiments are repeated after ∼5 min interval. (B and C) Time courses of locomotion speed when stimulating (B) the ILSC and (C) the DLSC for 5 s and then both sites (Sync) for 5 s. Top: Heatmaps of 12 individual trials from 4 mice. Bottom: Averaged locomotion speeds versus time. The solid lines and shaded areas indicate the mean and s.e.m., respectively. (D and E) Quantitative analyses of locomotion speed. Results are averaged in 5 s in different time courses. (D) before, during ILSC (5 s), during Sync (5 s), and after stimulations. (E) before, during DLSC (5 s), during Sync (5 s), and after stimulations. The statistical analysis method is one-way repeated measures ANOVA for data after logarithmic transformation (*n* = 12 trials, Sidak's multiple comparisons test, ∗∗∗p <0.001, ∗∗p <0.01, ∗p <0.05, n.s. p >0.05). Values are represented as mean ± s.d.

Another unique characteristic of our dual-channel micro-LED probes is the capability to activate different brain regions simultaneously, allowing the study of hierarchical structures of brain dynamics. With implanted micro-LED probes, we apply synchronized optical stimulations in the ILSC and the DLSC, to understand the inherent relationship between the two distinct defensive behaviors (freezing and flight). Figure 3A presents representative animal responses under stimulations in the ILSC and the DLSC (5-s duration; 20-Hz frequency; 10-ms pulse duration; 5-mA LED current), respectively, followed by immediate synchronized stimulations in both regions (the same parameters, 5-s duration). Videos S4 and S5 provide visualized data, correspondingly. Independent stimulations in the ILSC and the DLSC arouse freezing and flight responses, respectively, which are in accordance with the results obtained in Figure 2. Interestingly, the immediately imposed synchronized stimulations induce similar flight behaviors in both cases. More data traces are presented in Figures 3B and 3C, and statistics are given in Figures 3D and 3E. Data traces for the control group (without ChR2) are presented in Figure S6. Similar to results in Figure 2, we select the first 3 trails of each subject for statistical analysis. We analyze averaged locomotion speeds with standard deviations within four different time courses (before stimulation, during ILSC or DLSC stimulation, during synchronized stimulation, and after stimulation, all with durations of 5 s). In both scenarios, synchronized stimulations in both regions cause significantly enhanced speeds, resulting in evident flight behaviors. These behavioral responses can be ascribed to the different functions of the ILSC and DLSC (De Franceschi et al., 2016; Evans et al., 2018). Based on prior studies in rodent models, the ILSC receives inputs from visual and auditory sources related to distant threats, whereas the DLSC mostly processes information from auditory and somatosensory sources associated with more closer threats (May, 2006). Facing closer and more dangerous threats, mice have a greater tendency to flee. Therefore, the results associated with the synchronized modulations accord with the innate defensive response choices. In addition, the animals' motion speeds are even higher than those in response to stimulations in the DLSC only. This phenomenon could be ascribed to the stronger optical irradiance power, the increased illumination volume generated by the synchronized stimulation and a prolonged state of fear.

Video S4. Representative video showing a freely behaving mouse implanted with the micro-LED probe and the head-mounted circuit, when providing optogenetic stimulation: 5 s in the ILSC followed by a 5 s in both ILSC and DLSC (sync), LED current 5 mA, frequency 20 Hz, and duty cycle 20%. Related to Figure 3

Video S5. Representative videos showing a freely behaving mouse implanted with the micro-LED probe and the head-mounted circuit, when providing optogenetic stimulation: 5 s in the DLSC followed by a 5 s in both ILSC and DLSC (sync), LED current 5 mA, frequency 20 Hz, and duty cycle 20%. Related to Figure 3

### Immunohistochemistry after stimulations in the ILSC and the DLSC

**Figure 4:**
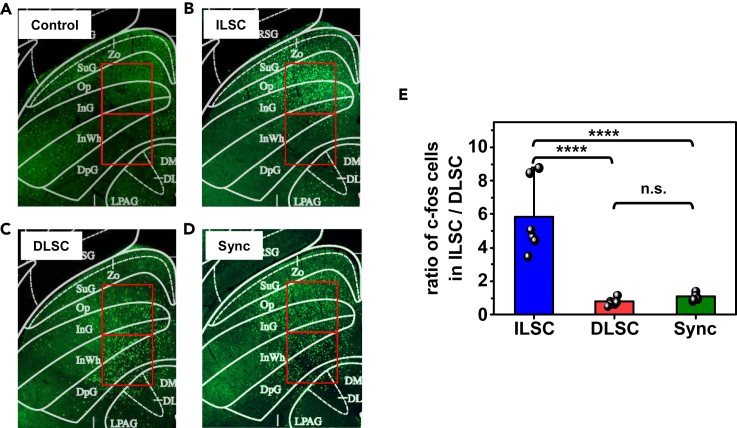
Results of the c-Fos staining in the SC after optogenetic stimulations in different modes (A–D) Representative fluorescence images of c-Fos activated neurons in the SC in different groups. (A) without stimulations (control). (B) stimulations in the ILSC. (C) stimulations in the DLSC. (D) stimulations in both sites (Sync). (E) Statistic analysis of the ratio of c-Fos activated neurons distributed in the ILSC and the DLSC through one-way ANOVA method (∗∗∗∗p <0.0001, n.s. p >0.05, *n* = 6 brain slices from 3 mice for each group).

Besides the analysis of induced defensive behaviors, we further investigate the effects of optogenetic stimulations on the ILSC and the DLSC by immunostaining of c-Fos, which has been previously found to be upregulated and is subjected to activations in these regions. Figures 4A–4D display the c-Fos expression patterns produced by optogenetic stimulations in different regions: (a) without any optogenetic stimulations (control), (b) only stimulating the ILSC, (c) only stimulating the DLSC, and (d) with synchronized stimulation in both regions (Sync). Compared to the control group, more c-Fos positive cells are observed in the ILSC and the DLSC upon illuminations. We analyze the ratio of numbers of cells expressing c-Fos in the ILSC and the DLSC (in a square area with a length of 610 μm each) (Figure 4E). Subjected to the stimulation in the ILSC, a dramatically larger number of c-Fos positive cells are found in the ILSC relative to that in the DLSC. By contrast, numbers of c-Fos cells in the ILSC and DLSC are similar (with a ratio of nearly 1:1) after stimulation is performed in the DLSC. Similar results are observed for the group after synchronized stimulations.

## Discussion

As an essential structure in the rodent brain, the SC not only mediates sensorimotor functions but also controls the defensive behaviors. The dual-channel micro-LED probe we have introduced here provides a viable means for interrogating the complex structures and functions in this region. In particular, the wireless optogenetic system has enabled systematic studies in mice's responses subjected to alternated and synchronized stimulations in the ILSC and the DLSC, which is previously inaccessible via conventional fibers with a single-spot emission. Table S1 summarizes operational characteristics of different multi-channel optogenetic systems reported in recent years (Ayub et al., 2017; Keppeler et al., 2020; Kim et al., 2013; Mayer et al., 2019; McAlinden et al., 2019; Park et al., 2015; Rossi et al., 2015; Wu et al., 2015; Yang et al., 2021). Although devices with more channels and smaller LED dimensions are also developed, most of them are based on wired circuits for power supply and control. Compared with existing wireless multi-channel optogenetic systems, our design presents a smaller LED geometry and a longer communication distance. When more functions or more LED devices are incorporated, the required circuit module will be larger and heavier, and improved circuit design can be utilized to reduce the system dimension (Jablonski et al., 2021). In addition, wireless energy harvesting strategies can be explored to realize battery-free operation, though in this case the communication distance can be limited by the near-field inductive coupling design (Shin et al., 2017; Yang et al., 2021). Demonstrated in the same animals, precisely controlled stimulations in the ILSC and the DLSC trigger distinct defensive behaviors with suppressed and enhanced locomotion (freezing and flight), respectively. Furthermore, animal behaviors (Figure 3) and immunostaining analysis (Figure 4) responding to synchronized stimulations also implicate the hierarchical structures of interconnected neurons in these regions. The facts that (1) synchronized stimulations evoke flight behaviors similar to the stimulation in the DLSC, and (2) stimulation in the DLSC also evokes upregulation of c-Fos expression in the ILSC indicate that neurons in the ILSC and the DLSC constitute different levels of control in the hierarchy of the SC, and different responses (freezing or flight) possess different priorities in the defensive behaviors. Admittedly, SC associated and defense relevant neural pathways are very complicated and cannot be fully unraveled using existing techniques. It is also noted that SC associated defensive responses are also greatly influenced by environments, for example, in the presence or absence of a nest (Wei et al., 2015; Shang et al., 2018). Behaviors in various scenarios could be studied in future explorations. One could also extend the use of such a device platform in the interrogation of other brain regions or nuclei. Unequivocally, further understanding the complicated brain structures and functions demands more advanced technologies based on dense arrays of microscale stimulators and detectors with high spatiotemporal resolutions and multiple functions, and the results presented here only provide a glimpse of what is possible from such a device strategy. Moving forward, multichannel microelectrodes (Fu et al., 2016; Xie et al., 2017), microfluidic channels (Jeong et al., 2015; Zhang et al., 2019), photometric (Lu et al., 2018), and electrochemical sensors (Liu et al., 2020) could be incorporated with our multi-channel optogenetic systems. To summarize, the multi-channel microscale optoelectronic device system will offer promising solutions to fundamental neuroscience research as well as biomedical applications.

### Limitations of the study

Although here we only perform behavior tests under constant light intensity and pulse frequency, the wirelessly operated, dual-channel probe enables versatile stimulation modes including varied stimulating times, frequencies, light intensities, and spatiotemporal sequences. Variable pulse frequencies and light intensities can further explore the threshold of the two different defensive behaviors caused by stimulating the two layers of SC (Evans et al., 2018). In this work, CaMKII promoter is used to express ChR2 in neurons in the SC, and a more desirable design can distinguish the roles of different cell types involved in the two defensive behaviors using transgenic mice. In addition, the near-Lambertian emission profiles from the micro-LEDs as well as the brain tissue scattering cause broad light distributions within the SC, which could leak to other regions such as the optical layer in the SLSC and cause possible phosphene. Therefore, further experiments are needed to validate the specificity of the stimulation of ILSC affecting behaviors. Smaller LEDs or collimated emitters (like lasers) can be employed to reach higher spatial resolutions. Moreover, multi-channel electrophysiological recordings could help record the activities and understand the relationship of neurons at different depths when optically stimulating neurons in ILSC or DLSC.

## STAR★Methods

### Key resources table

**Table:** 

REAGENT or RESOURCE	SOURCE	IDENTIFIER
**Antibodies**		

c-Fos (9F6) Rabbit mAb	Cell Signaling Technology	Cat# 2250; RRID: AB_2247211
Anti Iba1, Rabbit (for Immunocytochemistry)	WAKO	Cat# 019-19741; RRID: AB_839504
Anti-Glial Fibrillary Acidic Protein Antibody	Sigma-Aldrich	Cat# AB5541; RRID: AB_177521
Alexa Fluor 488-AffiniPure F(ab')2 Fragment Donkey Anti-Chicken IgY (IgG) (H+L)	Jackson ImmunoResearch Labs	Cat# 703-546-155; RRID: AB_2340376
Donkey anti-Rabbit IgG (H+L) Highly Cross-Adsorbed Secondary Antibody, Alexa Fluor 647	Thermo Fisher Scientific	Cat# A-31573; RRID: AB_2536183
IgG (H+L) Highly Cross-Adsorbed Donkey anti-Rabbit, Alexa Fluor™ 488	Thermo Fisher Scientific	Cat# A-21206; RRID: AB_2535792

**Bacterial and virus strains**		

AAV2/9-mCaMKIIa-mCherry-WPRE-pA	Shanghai Taitool Bioscience Co.Ltd.	S0242-9-H20
AAV2/9-mCaMKIIa-hChR2(H134R)-mCherry-WPRE-pA	Shanghai Taitool Bioscience Co.Ltd.	S0166-9-H50

**Experimental models: Organisms/strains**		

Mouse: C57BL∕6N (male, 8–12 weeks)	Vital River Laboratory	C57BL∕6NCrl

**Software and algorithms**		

Bonsai	Lopes et al., 2015	RRID: SCR_017218
Matlab R2018a	MathWorks	RRID: SCR_001622
IBM SPSS Statistics 25.0	IBM® SPSS® software platform	RRID: SCR_002865

### Resource availability

#### Lead contact

Further information and requests for resources and reagents should be directed to and will be fulfilled by the lead contact, Xing Sheng (xingsheng@tsinghua.edu.cn).

#### Materials availability

This study did not generate new unique reagents.

### Experimental model and subject details

#### Animal studies

All animal procedures were approved by the Institutional Animal Care and Use Committee (IACUC) at Tsinghua University. Adult (8–12 weeks) male C57BL/6N mice purchased from the Vital River Laboratory (Animal Technology, Beijing, China) were used and housed in groups (3–5 mice per cage) under standard conditions. Mice were maintained on a 12  h light/dark cycle (light on: 19:00; light off: 7:00) at 22–25°C and the behavior test were performed during the light phase of the cycle.

### Method details

#### Device fabrication

Detailed fabrication process is listed in Figure S1. The fabrication of micro-LED probes starts with adhering flexible substrates (PI/Cu/PI, 18/25/18 μm) on glass spin-coated with a layer of PDMS, with an additional layer of PI (∼10 μm) coated for insulation. The first blue micro-LED is transferred onto the substrate using an adhesive layer, with lithographically defined metal wires (Cr/Au/Cu/Au, 8 nm/100 nm/600 nm/150 nm) deposited for interconnection. An epoxy layer (SU8-3005, ∼5 μm) is applied to bond the second blue micro-LED, with interconnected metal wires patterned subsequently. The two-step transfer process and stacked electrode design reduce the probe width. Laser milling defines the needle shape of the implantable probe. Layers of PDMS (∼20 μm) and parylene-C (∼15 μm) that have better water-proofness and biocompatibility serve for final waterproof encapsulation.

#### Circuit design

The wireless circuit system (Figure 1F) includes a transmitter, a receiver and an injectable probe. The transmitter comprises a microcontroller (STM32F103C8T6) and a wireless transceiver module (nRF24L01P), programmed by a personal computer. The receiver includes a radio frequency (RF) transceiver (nRF24LE1, Nordic Semiconductor) to process RF signal and program the LED pulse frequency, pulse width and stimulation duration based on the received commands, two constant current drivers (ZLED7012) to drive the red indicating LEDs and micro-LEDs on the injectable probe with tunable current levels ranging from 1.8 mA to 20 mA independently. The receiver unit is formed as a detachable flexible printed circuit (FPC) powered by a lithium battery. The injectable probe is connected to the receiver circuit with a 4 pin FPC connector (Figure 1H), and the wearable part weights 1.7 g (FPC/battery, 0.5 g/1.2 g).

#### Optical modeling

A Monte-Carlo ray tracing method is employed to simulate the light propagation in the brain tissue (TracePro trial version). The device model includes two blue micro-LEDs with a distance of 610 μm and inserted into a tissue block of 8 mm × 8 mm × 8 mm. In the model, the brain tissue has an absorption coefficient of 0.3/mm, a scattering coefficient of 11.8/mm, an anisotropy factor of 0.85, and a refractive index of 1.36 at the wavelength of 470 nm. 2 million rays are traced to obtain the light distribution of the plane perpendicular to the surface of micro-LEDs. Rays emit randomly from micro-LEDs’ surface and obey the Lambertian distribution.

#### Thermal measurement and modeling

Temperature distributions on the surface of micro-LED probes (Figure S3) are measured using an infrared thermal camera (FOTRIC 220). The micro-LED probe is inserted into a brain phantom (made by 0.5% agarose, 1% intralipid and 0.25% bovine hemoglobin blood) underneath the surface by ∼300 μm. For calibration, the emissivity values of the probe surface and the brain phantom are set to 0.95 and 0.97, respectively. 3D steady heat transfer models are established by finite element analysis (COMSOL Multiphysics, heat transfer module) to simulate the temperature distributions. The parameters of the brain model include a density of 1.046 g/cm3, a thermal conductivity of 0.5 W/m/K, and a heat capacity of 3630 J/kg/K. The two micro-LEDs serve as the heat sources, with an input thermal power estimated by *P* = *V* × *I* × (1−*EQE*), where *V*, *I* and *EQE* are the measured voltage, current, and corresponding external quantum efficiency (∼12%) for LEDs. The material of the probe used for simulation is based on the device structure, including copper and polyimide substrate, GaN-based LEDs, SU-8 insulating layers and PDMS/parylene-C encapsulation layers.

#### Viral injection and probe implantation

Mice were anesthetized by intraperitoneal injection of 0.5% sodium pentobarbital (10  mL/kg), then shaved the scalp and placed in a stereotaxic frame. After adjusting the skull parallel to the reference panel, a hole with a diameter of ∼0.5 mm was drilled with a micromotor drill on the skull. Virus (150 nL) of AAV2/9-mCaMKIIα-hChR2(H134R)-mCherry for experimental groups or AAV2/9-mCaMKIIα-mCherry for control groups (both purchased from Shanghai Taitool Bioscience Co. Ltd) was slowly injected into the DLSC (AP:−3.72 mm, ML:±0.6 mm, DV −2.05 mm) and ILSC (AP:−3.72 mm, ML: ±0.6 mm, DV:−1.45 mm) with a speed of 20 nL/min. The needle was maintained in position for an additional period of 5 min before slow withdrawal to allow viral particles to diffuse and be absorbed at the injection site, and then we sutured the wound. After 3 weeks of virus expression, micro-LEDs were implanted with a similar procedure (Figure S4), into the SC (AP:−3.70 mm, ML:±0.6 mm, DV:−2.25 mm) slowly, then fixed the probe on the skull using dental cement.

#### Behavior test

Behavior test was carried out one week after probe implantation in an arena (30 cm × 30 cm), and a camera was used to record the mice activities. Before optogenetic stimulations, mice were allowed to explore the arena for 5 min to adapt to the environment. The shallower micro-LED stimulates the ILSC and the deeper micro-LED stimulates the DLSC, with a frequency of 20 Hz, a pulse width of 10 ms, and an injection current of 5 mA. We performed ILSC stimulation (3 s) and DLSC stimulation (3 s) alternately with 5-min interval for 10 times (total 20 trials, 100 min) for mice analyzed in Figure 2. Tests for synchronized stimulations were performed with similar parameters in Figure 3 (5-s ILSC + 5-s sync and 5-s DLSC + 5-s sync, with 5-min interval for 10 times, total 10 trials, 100 min). Animals' locomotion was analyzed from the recorded videos through the software Bonsai and customized MATLAB codes. The locomotion speed was calculated by the mice moving distance difference per 0.5 s. For c-Fos experiments, no stimulation, ILSC, DLSC or synchronized stimulations were applied for different groups of mice, separately (5 s, 10 times, 3-min interval between each stimulation), and then mice were sacrificed 1.5 h after these stimulations.

#### Immunohistochemistry

Mice were anesthetized with 0.5% sodium pentobarbital (10  mL/kg) and perfused intracardially with phosphate buffer saline (PBS) solution and 4% paraformaldehyde. The brains were post fixed in 4% paraformaldehyde overnight at 4°C, and then frozen sectioned into slices with a thickness of 40 μm. The sections are washed with phosphate buffered saline (PBS, pH = 7.4) and PBST (0.2% Triton X-100 in PBS), and then incubated with blocking solution (3% BSA in PBST) for 1–2 h at room temperature. The slices were then incubated with the following primary antibodies at 4°C for 24–48 h: c-Fos (9F6) Rabbit mAb (1:200) for indicating activated neurons, anti-Iba1 (1:1000, rabbit) and anti-GFAP (1:1000, chicken) for indicating microglia and astrocytes, respectively. Following three times rinsed (10 min each) in the PBST solution, slices were incubated with the secondary antibodies (donkey anti-rabbit IgG Antibodies and donkey anti-chicken IgY antibodies) and DAPI for 2 h at room temperature avoiding light. Then sections were washed five times with PBS solution and mounted on slides with antifade mounting medium (VECTASHIELD, Cat. No. H-1200). The images of the immunohistochemistry were captured on ZEISS Axio Scan.Z1 and Zeiss LSM 710 Meta.

### Quantification and statistical analysis

All data were analyzed with IBM SPSS Statistics Software, and details of the statistical test are described in the figure legends. ANOVA method followed by Sidak's multiple comparisons test was performed. Statistical significance was designated for analyses with p <0.05. Asterisks in all figures indicate the degree of significant differences compared to controls, n.s., p > 0.05; ∗, p <0.05; ∗∗, p <0.01; ∗∗∗, p <0.001; ∗∗∗∗, p <0.0001.

## Data Availability

• No dataset was generated from or used in this study. • No original code was reported in this study. • Additional information related to this study will be fulfilled by the lead contact upon request.

## Supplemental information

Document S1. Figures S1–S6

Table S1. Comparison of operational characteristics among different multi-channel optogenetic systems, Related to Figure 1
