# Supplementary material for: A dual-channel optogenetic stimulator selectively modulates distinct defensive behaviors
Source: iScience. 2021 Dec 24;25(1):103681. doi: 10.1016/j.isci.2021.103681 (PMC8749196; doi:10.1016/j.isci.2021.103681)
Supplement: Document S1. Figures S1–S6 [file mmc1.pdf]

iScience, Volume *25*

Supplemental information

A dual-channel optogenetic stimulator selectively modulates distinct defensive behaviors

Xue Cai, Lizhu Li, Wenhao Liu, Nianzhen Du, Yu Zhao, Yaning Han, Changbo Liu, Yan Yin, Xin Fu, Dawid Sheng, Lan Yin, Liping Wang, Pengfei Wei, and Xing Sheng

Figure S1

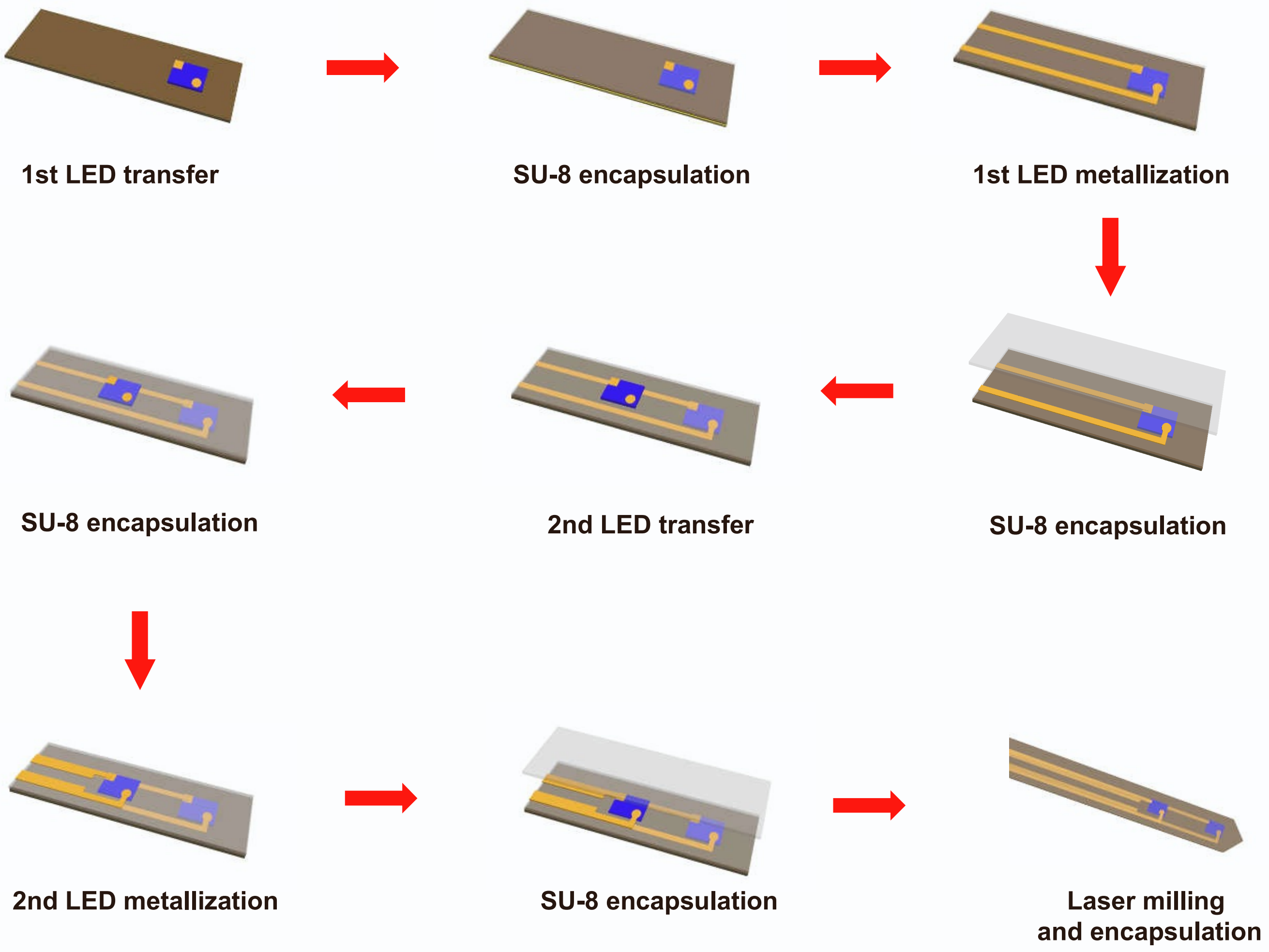

Figure S1. Schematic illustration for manufacturing the dual-channel micro-LED probe. Related to Figure 1.

Figure S2

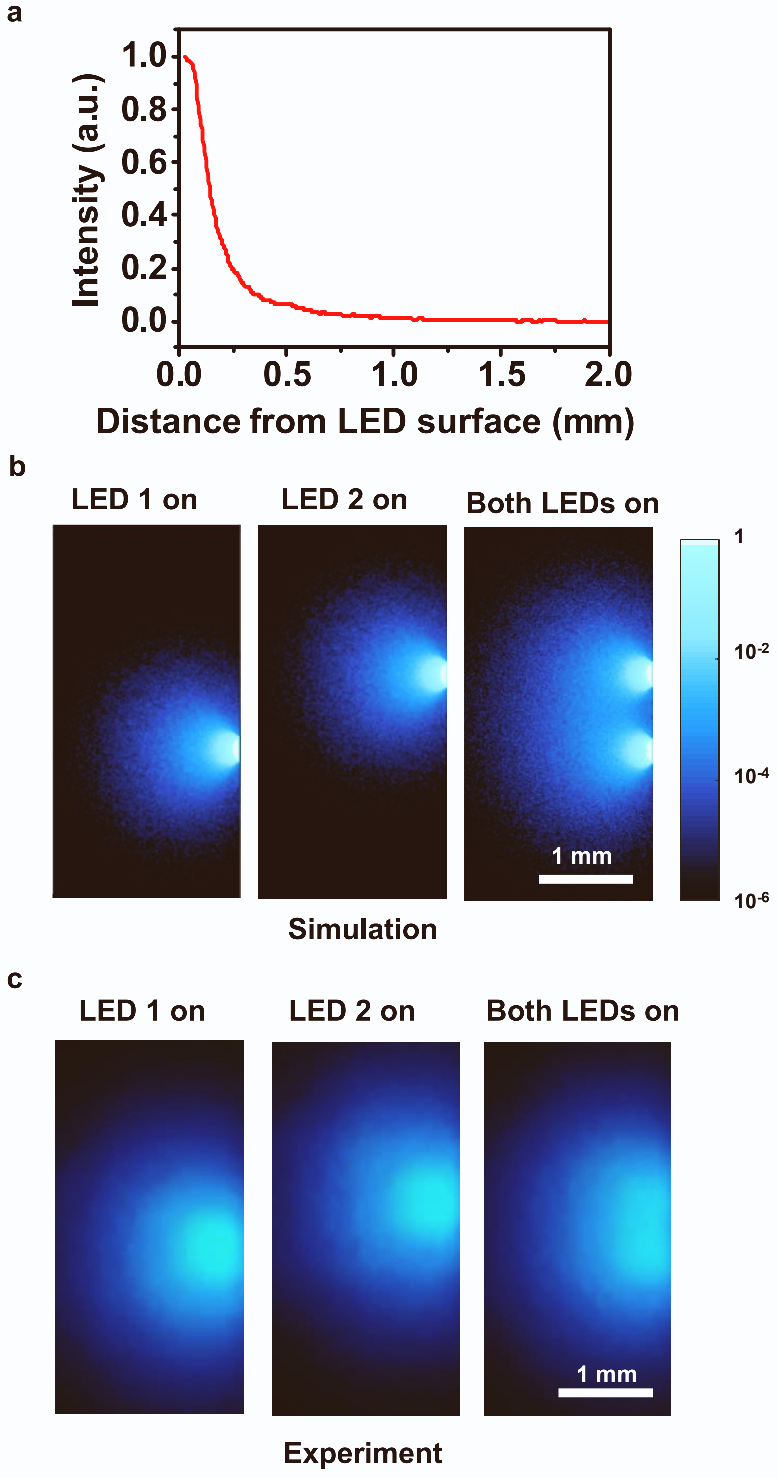

Figure S2. Light distribution in the tissue. Related to Figure 1. **(a)** Simulated relative light intensity distribution in the brain tissue as a function of distance from a micro-LED surface. **(b)** Simulated light distribution in brain tissues when LED1 is on, LED2 is on, and both LEDs are on. **(c)** Optical graphs of light distribution in brain tissue phantoms with an inserted probe when LED1 is on, LED2 is on, and both LEDs are on. The quality of these photographs is limited by the signal sensitivity of the camera.

Figure S3

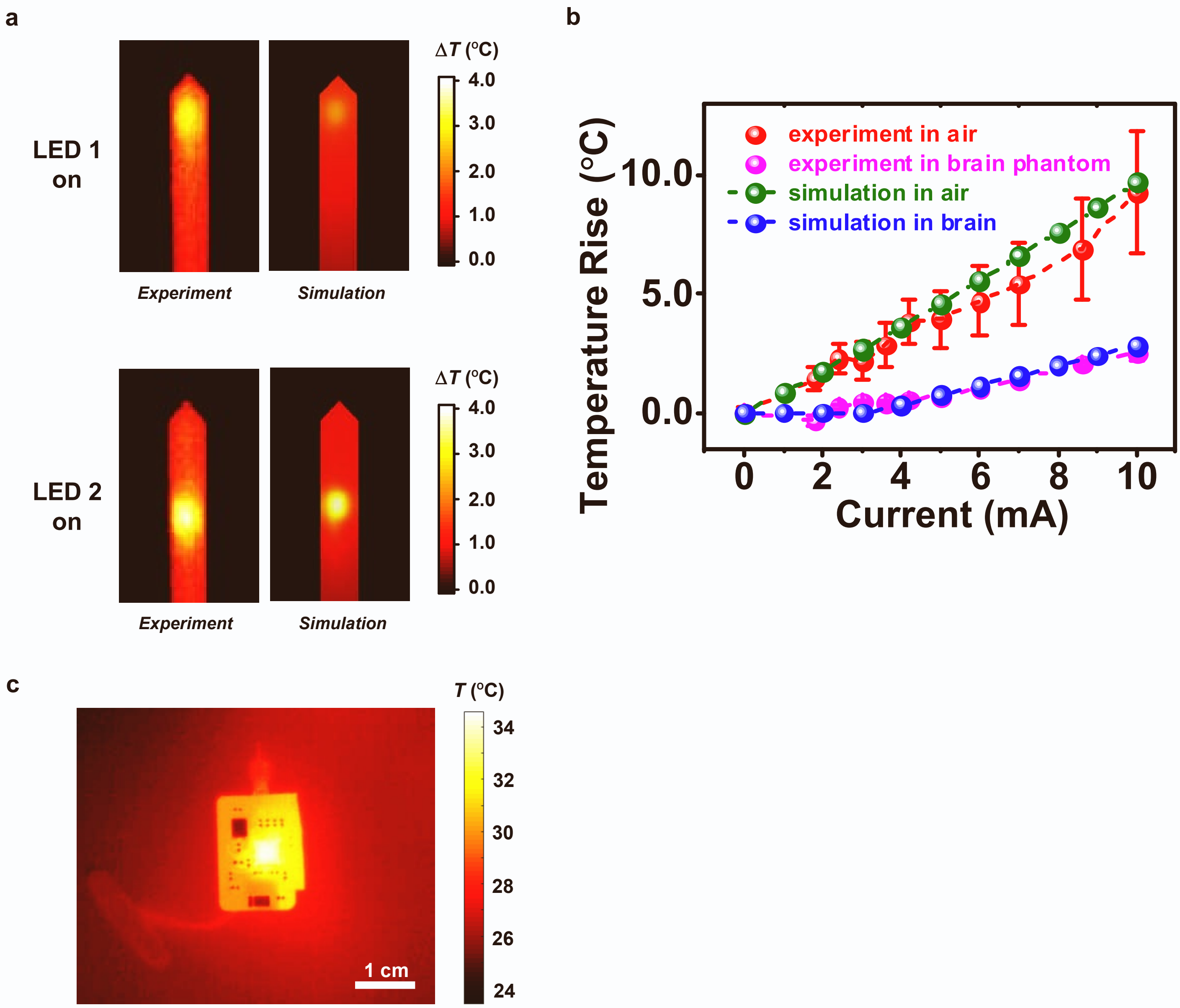

Figure S3. Thermal properties of the probe. Related to Figure 1. **(a)** Measured (left) and simulated (right) temperature distributions on the probe when the first or the second LED is operated in air (LED current 5 mA, frequency 20 Hz, duty cycle 20%). **(b)** Measured and simulated maximum temperature rises above room temperature on the top surface of the probe as a function of pulsed currents (frequency 20 Hz and duty cycle 20%, both LEDs are on). Measurements are performed in air and a brain phantom, and simulations are perform in air and in the brain tissue. **(c)** Measured temperature distribution on the top surface of the wireless circuit during operation.

Figure S4

a b c d

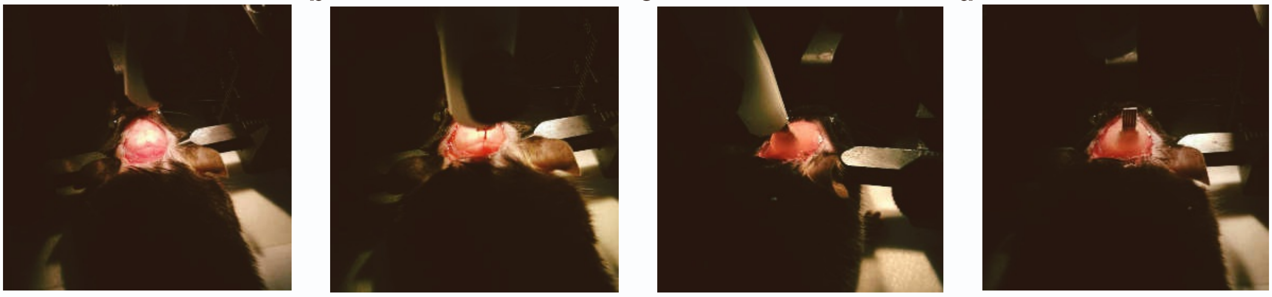

Figure S4. The procedure of implanting the dual-channel microprobe. Related to Figure 1. **(a)** cutting the scalp and exposing the skull. **(b)** drilling a hole on the skull and implanting the probe into mouse brain. **(c)** applying the dental cement to fix the probe to the skull. **(d)** Loosening the clamp after the dental cement drying.

Figure S5

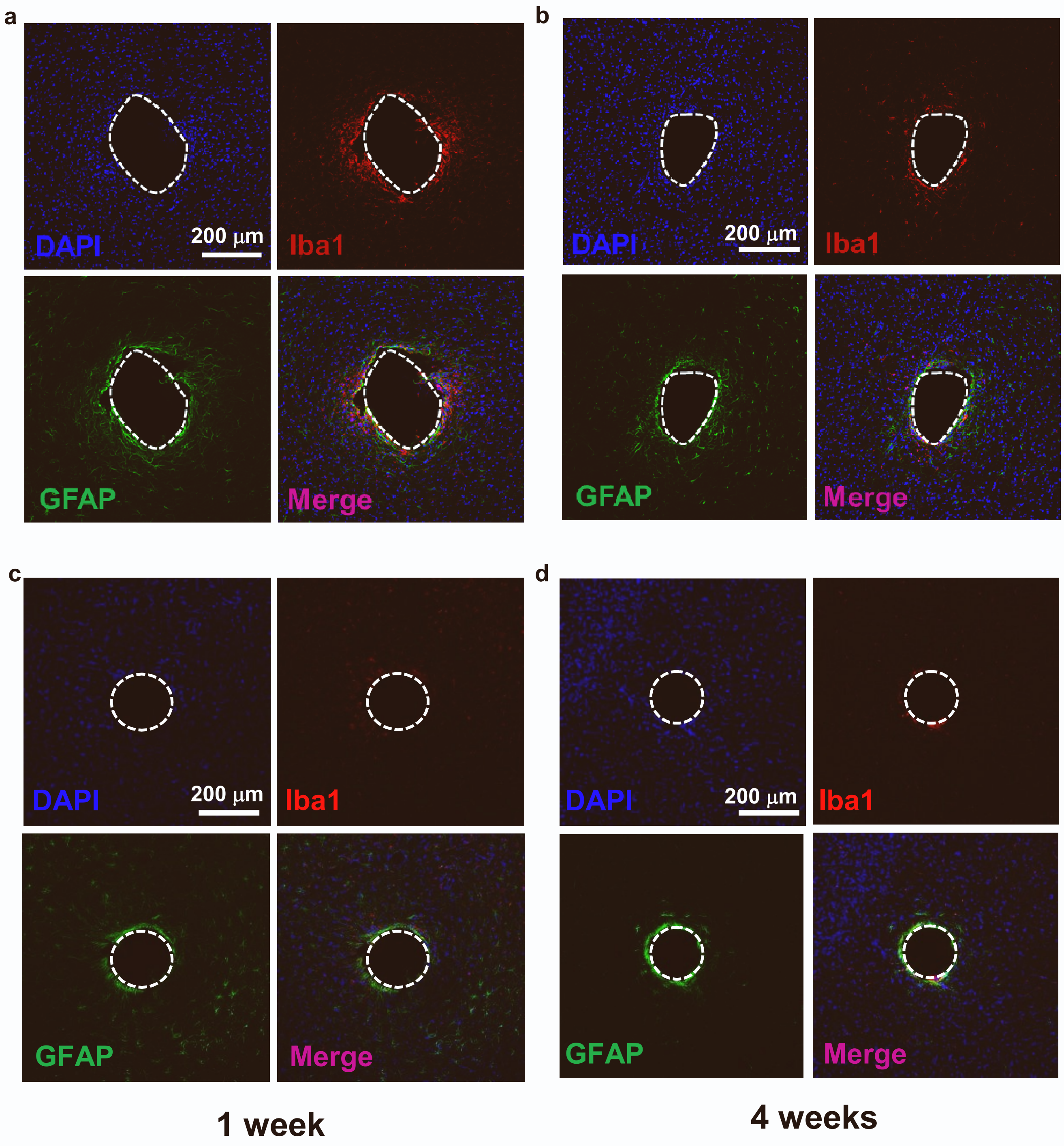

Figure S5. Inflammatory reactions triggered by implanting microprobe. Related to Figure 2. Representative confocal fluorescence images show immunohistochemical staining for DAPI (blue), activated microglia (Iba1, red), astrocytes (GFAP, green) and the merged images **(a, b)** after probe implantation for 1 week and 4 weeks, and **(c, d)** after silica fiber implantation for 1 week and 4 weeks. Lesion areas are outlined in white dashed lines. Differences in (a) and (b) indicate that the probe dimension has some deviations from the design (width ~310 μm and thickness ~150 μm) due to the variations of laser milling and encapsulation (10~50 μm). In addition, some acute trauma can partially recover after a long time (~4 weeks), and the tissue collapse after removing the probe also contributes to the reduction of the lesion area in (b).

Figure S6

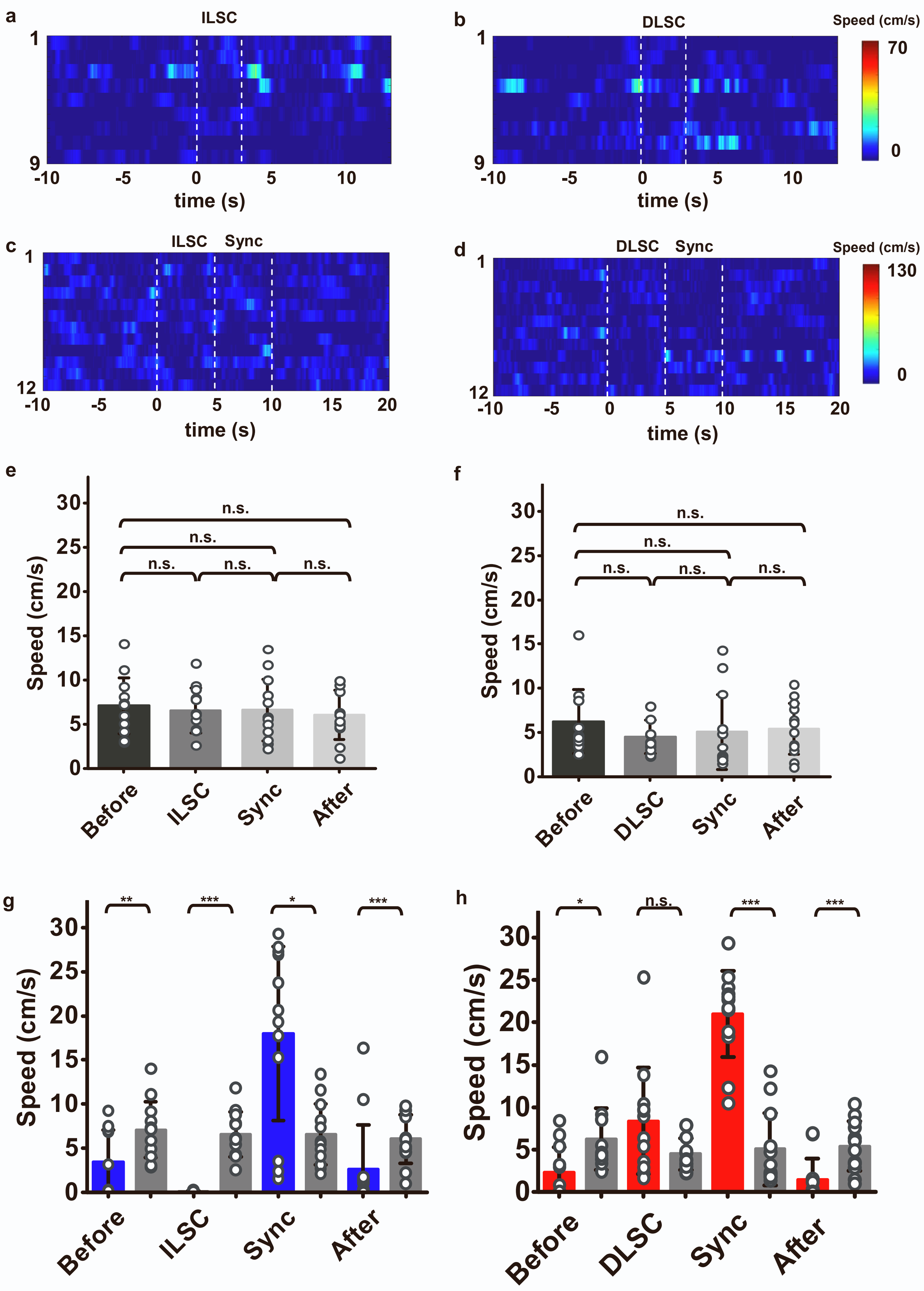
a
ILSC
1
9
-10
-5
0
5
10
time (s)
b
DLSC
Speed (cm/s)
70
0
time (s)
c
ILSC
Sync
1
12
-10
-5
0
5
10
15
20
time (s)
d
DLSC
Sync
Speed (cm/s)
130
0
time (s)
e
Speed (cm/s)
n.s.
Before
ILSC
Sync
After
f
Speed (cm/s)
n.s.
Before
DLSC
Sync
After
g
**
***
*
***
Speed (cm/s)
Before
ILSC
Sync
After
h
*
n.s.
***
***
Speed (cm/s)
Before
DLSC
Sync
After

Figure S6. Analyses of locomotion speed of control groups under stimulations of intermediate, deep layers (ILSC and DLSC) and ILSC or DLSC followed by synchronized stimulations in both sites (Sync). Related to Figures 2 and 3. **(a, b)** Heatmaps of 9 individual trials from 3 control mice (a) before, during ILSC (3 s) and after stimulations. (b) before, during DLSC (3 s) and after stimulations. **(c, d)** Heatmaps of 12 individual trials from 4 mice. Time courses of locomotion speed when stimulating (c) the ILSC and (d) the DLSC for 5 s and then both sites (Sync) for 5 s. **(e, f)** Quantitative analyses of locomotion speed. Results are averaged in 5 s in different time courses. (e) before, during ILSC (5 s), during Sync (5 s) and after stimulations. (f) before, during DLSC (5 s), during Sync (5 s) and after stimulations. The statistical analysis method is one-way repeated measures (n = 12 trails, Sidak's multiple comparisons test). **(g, h)** Quantitative analyses of locomotion speed between the experimental group and the control group. The statistical analysis method is two-way repeated measures ANOVA (n = 12 trails for Exp, n = 12 trails for Ctrl, Sidak's multiple comparisons test). *** $P < 0.001$, ** $P < 0.01$, * $P < 0.05$, n.s. $P > 0.05$). Values are represented as mean ± s.d..
